# Predictive value of an ultrasound-based radiomics model for central lymph node metastasis of papillary thyroid carcinoma

**DOI:** 10.7150/ijms.95022

**Published:** 2024-06-24

**Authors:** Weina Jia, Yundan Cai, Shu Wang, Jianwei Wang

**Affiliations:** 1 Department of Ultrasound, The Second Affiliated Hospital of Zhejiang Chinese Medical University, Hangzhou 310000, Zhejiang Province, China.; 2 Department of Ultrasound, Shanghai Sixth People's Hospital Affiliated to Shanghai Jiao Tong University School of Medicine, Shanghai 200233, China.; 3 Department of Ultrasound Diagnosis and Treatment, Xi'an International Medical Center Hospital, Xi'an 710100, Shaanxi Province, China.

**Keywords:** diagnosis, lymph nodes, metastasis, thyroid cancer, papillary, ultrasonography.

## Abstract

**Purpose:** We aimed to explore the predictive value of an ultrasound-based radiomics model for the central lymph node metastasis of papillary thyroid carcinoma.

**Methods:** A total of 126 patients with papillary thyroid carcinoma treated between February 2021 and February 2023 were retrospectively enrolled and assigned into metastasis group (n=59, with cervical central lymph node metastasis) or non-metastasis group (n=67, without metastasis) based on surgical and pathological findings. Intergroup comparisons were conducted on the results of contrast-enhanced ultrasonography, preoperative conventional ultrasonography, as well as real-time shear wave elastography.

**Results:** The maximum lesion diameter, echo, margin, capsule invasion, calcification, average elasticity modulus (Eavg), rising time (RT), and peak intensity (PI) had diagnostic value for papillary thyroid carcinoma, and their combination exhibited higher diagnostic value (area under the curve: 0.817). The logistic regression model was built, and the maximum lesion diameter, hypoechoic/extremely hypoechoic, lobulated or irregular margin (95% confidence interval: 1.451-6.755), capsule invasion, microcalcification/macrocalcification or peripheral calcification, high-level Eavg, low-level RT and high-level PI served as risk elements affecting papillary thyroid carcinoma from the aspect of central lymph node metastasis (odds ratio>1, P<0.05). According to the logistic regression model, the model was reliable and stable (area under the curve: 0.889, P<0.05).

**Conclusion:** The established ultrasound-based radiomics model can be utilized for early identifying the central lymph node metastasis of papillary thyroid carcinoma.

## Introduction

Papillary thyroid carcinoma can invade the surrounding organs, compressing the trachea and esophagus and inducing symptoms such as dyspnea and dysphagia 1. In addition, it may metastasize if no effective treatment is carried out promptly, then endangering the life and safety of patients 2. Despite slow growth, papillary thyroid carcinoma is extremely prone to central lymph node metastasis 3. As a risk influencing papillary thyroid carcinoma in terms of relapse besides distant metastasis, central lymph node metastasis has a severe adverse effect on patients' quality of life and survival rate 4,5. In the past, the sufferers of papillary thyroid carcinoma were usually treated by prophylactic central lymph node dissection to improve the long-term prognosis 6. However, the American Thyroid Association (ATA) pointed out in 2015 that in clinical practice, lymph node dissection is not applicable to node-negative patients with papillary thyroid carcinoma, so as to reduce iatrogenic damage 7. Therefore, accurately identifying the pathological state of lymph nodes in the central cervical region of patients before surgery is of great significance for the formulation of targeted therapeutic regimens.

Currently, the central lymph node metastasis of papillary thyroid carcinoma is mainly diagnosed with conventional ultrasonography. The number and size of cervical lymph nodes can be preliminarily analyzed based on suspected ultrasound features of lymph node metastasis involving hilum disappearance, calcification, internal cystic change and unclear margin 8. However, conventional ultrasonography alone has a high rate of missed diagnosis and limited ability to identify central lymph node metastasis before surgery because of the lymph nodes located deeply in the central region plus the interference of factors such as cervical and tracheal artifacts. In contrast, multi-mode ultrasonography exhibits obvious advantages in reflecting the features and adjacent relationship of thyroid tumors 9.

Lambin *et al.* proposed the concept of "radiomics" in 2012. They extracted and screened the advanced features of images through high-throughput analysis, which can deeply explore the characteristics of diseases based on conventional imaging diagnosis 10. Then Doroshow *et al.* applied radiomics to ultrasound examination, and comprehensively evaluated the nature and degree of disease using multiple modes and parameters 11. This method combines algorithms to transform image data into pixel data and feature space to create mathematical models or data sets, which is conducive to improving the diagnostic efficiency.

Given this, in this study, contrast-enhanced ultrasonography (CEUS), conventional ultrasonography as well as real-time shear wave elastography (SWE) were conducted on subjects suffering from papillary thyroid carcinoma, and an ultrasound-based radiomics model was built to predict the central lymph node metastasis for the first time, aiming to provide valuable evidence for clinical diagnosis and treatment.

## Methods

### Collection of general data

This study was approved by the ethics committee of our hospital, and written informed consent was obtained from all patients. A total of 126 patients suffering from papillary thyroid carcinoma, who received treatment in the hospital during February 2021 and February 2023, were retrospectively selected and assigned into metastasis group (n=59, with cervical central lymph node metastasis) and non-metastasis group (n=67, without metastasis) based on surgical and pathological findings. The general data in the two groups were comparable (P>0.05) (Table 1).

### Inclusion and exclusion criteria

The following inclusion criteria were used: (1) patients having thyroid nodules meeting the American Radiological Society Ti-RADS Class 3 and above standards based on conventional ultrasonography and receiving thyroid fine needle aspiration before examination^12^, (2) those scheduled to receive thyroidectomy, (3) those diagnosed with papillary thyroid carcinoma by pathological examination after surgery, (4) those with complete data of multi-mode ultrasonography within one month before surgery, and (5) those with unilateral lesions.

The exclusion criteria involved: (1) patients receiving chemotherapy, radiotherapy, endocrine therapy or other anti-tumor treatments in the past year, (2) those with other types of thyroid carcinoma, (3) those complicated by primary tumors at other sites, (4) those allergic to ultrasound contrast agents or unable to undergo CEUS, or (5) those complicated with immune system diseases or blood system diseases.

### Method for ultrasonography

The patients were in a supine position, with hands at their sides naturally, and a soft pillow was put under the neck to make the neck hyperextended, thus fully exposing the neck. Then two senior ultrasound doctors performed multi-mode ultrasonography, and recorded and interpreted the results.

### Conventional ultrasonography method

**Figure 1:**
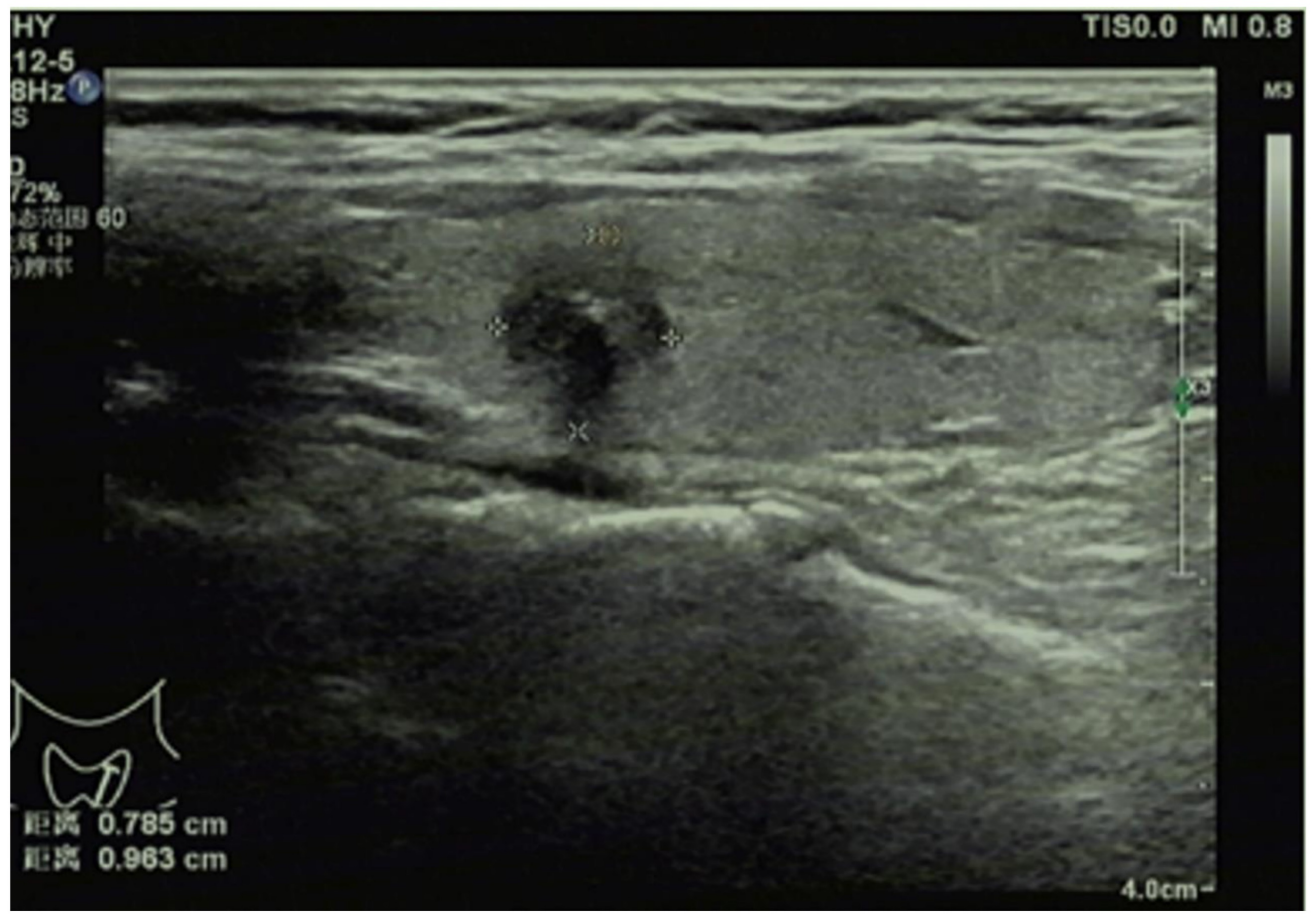
Conventional ultrasonography for papillary thyroid carcinoma.

Doppler ultrasound diagnostic instrument (LOGIQ E8, GE, USA) equipped with 4-18 MHz linear array probes was employed for conventional ultrasonography of thyroid glands and cervical lymph nodes (Figure 1). In brief, the bilateral lobes and isthmus of thyroid glands were scanned for the transverse section and then for the sagittal section from top to bottom, and the conventional ultrasonic image data were recorded in detail, including the shape, size, echo and blood flow of thyroid glands. After finding nodules, the following parameters were recorded: the maximum lesion diameter, number of nodules (single or multiple), aspect ratio of lesions (≥1 or <1), location (right lobe, left lobe, or isthmus), echo (hyperechoic or isoechoic, or hypoechoic/extremely hypoechoic), margin (smooth, lobulated or irregular), capsule invasion (yes or no), calcification (yes or no), and blood flow distribution (Grade 0, I, II, or III, assessed by Adler's blood flow grading standard, with Grade 0 for no blood flow signal in lesions, grade I for existence of 1-2 thin short rod-like/dot blood flow signals in lesions, Grade II for presence of 1 relatively long blood vessel or 3-4 thin short rod-like/dot blood flow signals in lesions, and Grade III for existence of 2 relatively long blood vessels or more than 5 thin short rod-like/dot blood flow signals in lesions). Thereafter, all-round scan of cervical lymph nodes was conducted, with a high frequency linear array probe for continuous scan from the submentum to the jaw.

### Real-time SWE method

**Figure 2:**
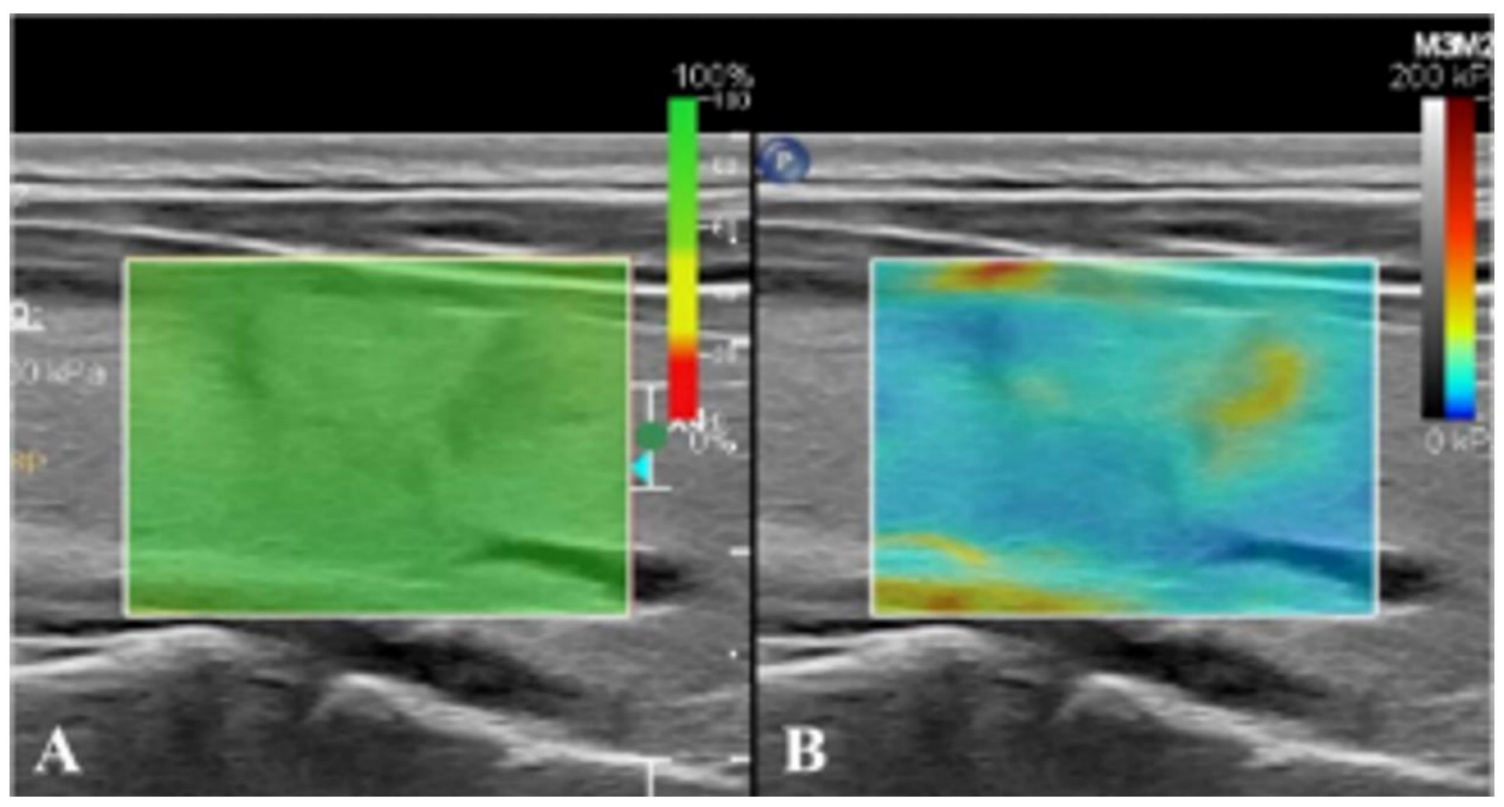
** Real-time SWE. (A)** SWE image with reliability of 100%. **(B)** SWE mode showing that the hardness of nodules is greater than that of normal glands at the same depth.

After conventional ultrasonography was completed, the real-time SWE mode was switched to observe the hardness of lesions and surrounding tissues. Specifically, a 2 mm sampling frame was used to select the affected nodules and surrounding normal gland tissues as the region of interest. When the sampling frame displayed stable color without mosaic color points, the timer was started, with images saved dynamically (Figure 2). Then the elastic modulus value in the sampling frame was measured by Q-Box measurement software (Panotec, Italy). Measurement was repeated five times on the same lesion and the same section to take the average elasticity modulus (Eavg) and the ratio to that of surrounding normal tissues (Eratio).

### CEUS method

**Figure 3:**
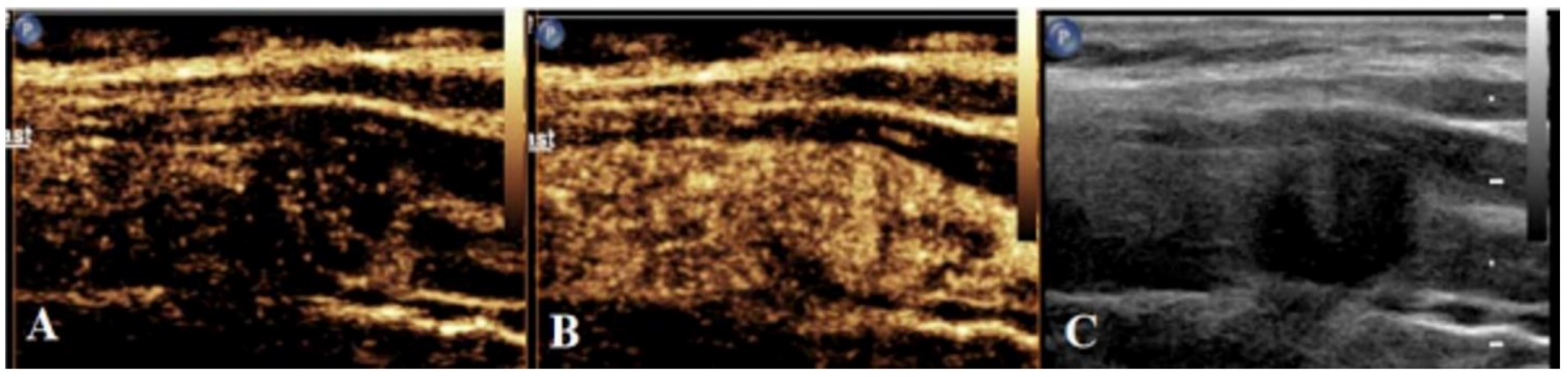
** Schematic diagram of centrifugal enhancement of nodules in CEUS mode. (a)** At 10 s after contrast agent injection, enhancement is firstly found in the center of nodules, followed by the periphery. **(b)** At 17 s after contrast agent injection, nodules display uneven iso-high enhancement. **(c)** Two-dimensional ultrasonic diagram of nodules during CEUS dual imaging.

After real-time SWE, CEUS was conducted as follows. The elbow vein was injected with contrast agent (2.4 mL; Sonova, Switzerland), followed by tube flushing with 5 mL of normal saline. Next, dynamic images were saved after starting the timer. CEUS was ended following continuous observation for a minimum of 90 s (Figure 3). The time-intensity curve (TIC) was plotted using contrast analysis software, for the purpose of recording time to peak (TTP), rising time (RT), area under TIC (AUCt) and peak intensity (PI).

### Construction of ultrasound radiomics model

The margin of thyroid lesions was manually outlined by two senior sonographers. Then the ultrasonic image data of lesions were analyzed by the radiomics method. The advanced features of images, including shape, gray-scale histograms, texture, and wavelet features, were extracted and screened by Pyradiomics software in a high-throughput manner 13. Afterwards, a prediction medical model was built using the physical algorithm of support vector machine.

### Evaluation of outcomes

The results of preoperative conventional ultrasonography, real-time SWE and CEUS in the metastasis group were compared with those of the non-metastasis group.

### Statistical analysis

SPSS 23.0 software (IBM Inc., USA) for statistical analysis was employed. The expression format of (‾*x* ± s) plus the *t*-test for analysis were used for measurement data, while count data were expressed by [n (%)] and subjected to the *χ*^2^ test. The value of conventional ultrasonography, real-time SWE and CEUS in diagnosing central lymph node metastasis of papillary thyroid carcinoma was investigated by drawing receiver operating characteristic (ROC) curves. A logistic regression model was established to explore the influencing factors for central lymph node metastasis. P<0.05 signified a statistically significant difference.

## Results

### Comparison of conventional ultrasonography results between metastasis and non-metastasis groups

Through comparison of the two groups, the maximum lesion diameter, echo, margin, capsule invasion and calcification were statistically significantly different (P<0.05), whereas the number of nodules, aspect ratio of lesions, location and blood flow distribution were comparable (P>0.05) (Table 2).

### Comparison of real-time SWE results between metastasis and non-metastasis groups

The metastasis group presented an increased Eavg by contrast to the non-metastasis group, and the disparity was statistically significant (P<0.05), while no statistically significant intergroup difference was found in the Eratio (P>0.05) (Table 3).

### Comparison of CEUS results between metastasis and non-metastasis groups

The metastasis group had a shorter RT and a higher PI in contrast with the non-metastasis group, presenting differences of statistical significance (P<0.05). No statistically significant differences were observed in TTP and AUCt by comparing the two groups (P>0.05) (Table 4).

### Diagnostic values of ultrasound imaging features/parameters for central lymph node metastasis

**Figure 4:**
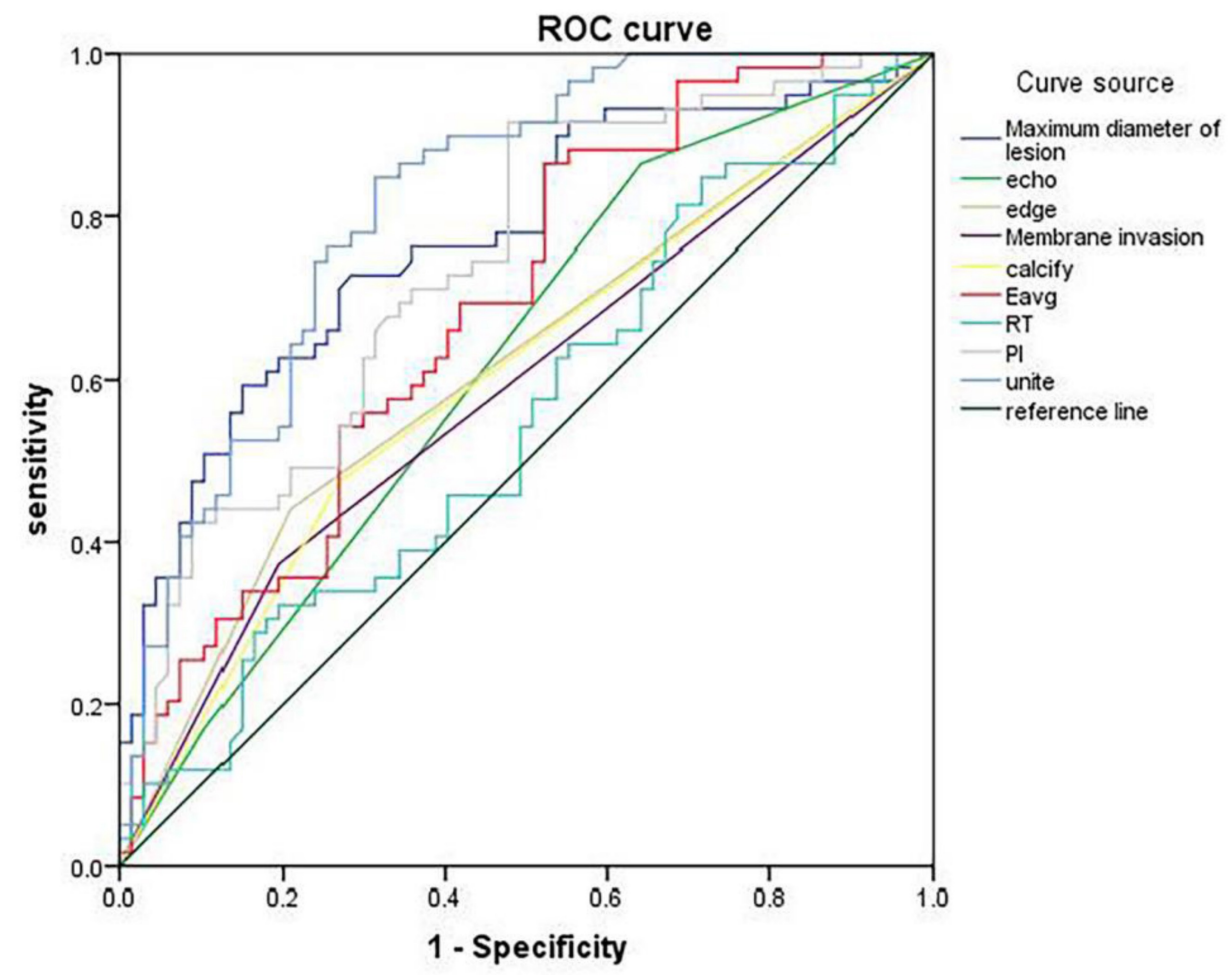
ROC curve of ultrasound imaging features/parameters in diagnosing central lymph node metastasis.

The ROC curves were plotted with the indicators with differences as the test variables and the central lymph node metastasis of papillary thyroid carcinoma as the state variable (1=metastasis, 0=non-metastasis) (Figure 4). Maximum lesion diameter, echo, margin, capsule invasion, calcification, Eavg, RT, and PI displayed diagnostic values for papillary thyroid carcinoma [area under curve (AUC)=0.773, 0.579, 0.616, 0.589, 0.603, 0.687, 0.551, and 0.740], and their combination had higher diagnostic value (AUC=0.817) (Table 5).

### Results of multivariate logistic regression analysis on central lymph node metastasis

A logistic regression model was built, with the conventional ultrasonography, real-time SWE and CEUS results that were different between the two groups as the independent variables, and the central lymph node metastasis in subjects experiencing papillary thyroid carcinoma as the dependent variable (1= metastasis, 0= non-metastasis). It was discovered that the maximum lesion diameter [95% confidence interval (CI): 1.193-1.535], hypoechoic/extremely hypoechoic (95% CI: 1.451-8.727), lobulated or irregular margin (95% CI: 1.451-6.755), capsule invasion (95% CI: 1.106-5.514), microcalcification/macrocalcification or peripheral calcification (95% CI: 1.169-5.172), high-level Eavg (95% CI: 1.033-1.112), low-level RT (95% CI: 0.950-1.214), and high-level PI (95% CI: 1.116-1.341) were risk elements influencing central lymph node metastasis (odds ratio>1, P<0.05) (Table 6).

### Validation of model prediction effect

**Figure 5:**
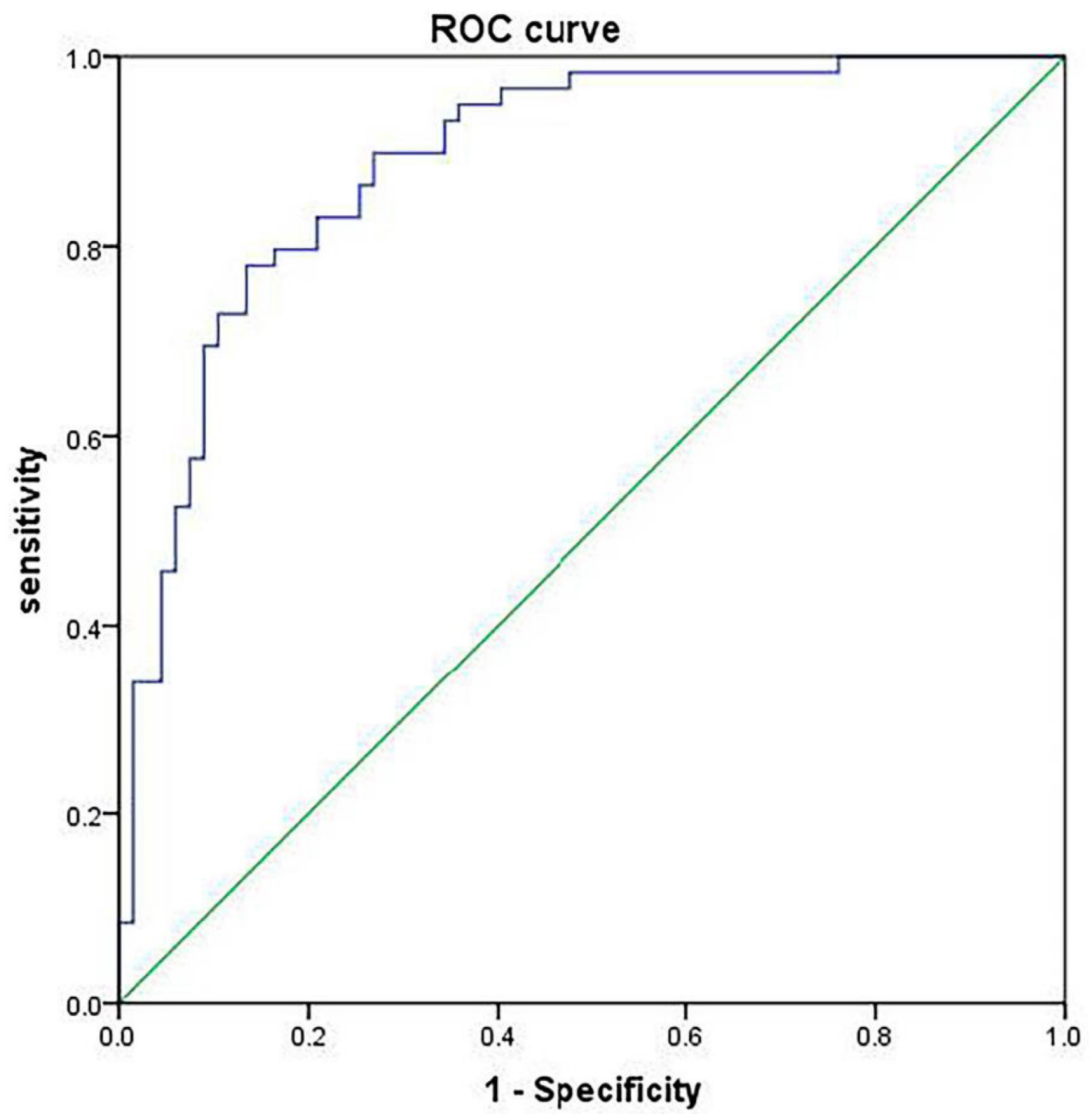
ROC curve for validation of model prediction effect.

According to the logistic regression model, the following regression equation was obtained: Logit(P)=2.320+1.323×maximum lesion diameter+3.558×echo+3.131×margin+2.470×capsule invasion+2.459×calcification+1.072×Eavg+1.074×RT+1.224×PI. With the calculated prediction probability as the test variable and the central lymph node metastasis of papillary thyroid carcinoma as the state variable (1=metastasis, 0=non-metastasis), a ROC curve was plotted, and it was found that AUC=0.889, suggesting that the model is reliable and stable (Table 7 and Figure 5).

## Discussion

As a common indolent malignancy of the endocrine system, papillary thyroid carcinoma is characterized by slow growth, but statistics showed that about half of patients with papillary thyroid carcinoma will experience lymph node metastasis, and even those with tiny papillary thyroid carcinoma with a diameter of ≤10 mm are no exception 14,15.

Central lymph node metastasis of papillary thyroid carcinoma does not affect the short-term survival of patients, but it serves as a leading factor for tumor recurrence and has a great impact on the long-term survival of patients 16,17. In the past, patients with papillary thyroid carcinoma were recommended to undergo surgical resection in combination with central lymph node dissection, but there was no evidence of substantial benefit. Moreover, some studies have pointed out that prophylactic lymph node dissection may lead to many complications, such as impairment of parathyroid function and recurrent laryngeal nerve injury, which will increase iatrogenic injury and harm the prognosis of patients 18,19.

Currently, ultrasound-guided needle biopsy serves as the gold standard for clinical diagnosis of central lymph node metastasis of papillary thyroid carcinoma, which, however, is an invasive examination and thus will damage the organs around the lesions to some extent, giving rise to such side effects as pain, bleeding and fever. As a result, its clinical application is restricted 20,21. At present, imaging examination is still the preferred choice for clinical examination of thyroid glands and lymph nodes. Conventional ultrasonography enables the selection of clear two-dimensional sections from multiple angles to observe the shape, margin, echo, internal structure and other information of lesions, which has high diagnostic sensitivity for papillary thyroid carcinoma 22,23.

In this study, conventional ultrasonography was applied to patients with papillary thyroid carcinoma. The results revealed that the maximum lesion diameter, echo, margin, capsule invasion and calcification in the metastasis group were significantly different from those in the non-metastasis group. Besides, it was confirmed through the logistic regression analysis that the maximum lesion diameter, hypoechoic/extremely hypoechoic, lobulated or irregular margin, capsule invasion, and microcalcification/macrocalcification or peripheral calcification were risk factors for central lymph node metastasis of papillary thyroid carcinoma. Papillary thyroid carcinoma typically manifests irregular shape, lobulated margin, hypoechoic or extremely hypoechoic, invasion of the adjacent capsule, microcalcification and so on. In 2015 ATA guidelines, extraglandular invasion is listed as a risk factor for recurrence of thyroid carcinoma 24. When papillary thyroid carcinoma has a large lesion diameter, lobulated or irregular margin and capsule invasion, the tumor is large and relatively highly invasive, with angiogenesis, which will increase the contact between the tumor and lymphatic vessels in glands, thus increasing the risk of lymphatic metastasis of tumor cells 25,26. Calcification mainly results from the liquefaction of cancer nodules or failures to excrete some calcified substances secreted by cancer tissues, which mostly occurs in solid and diffuse sclerosing variant of papillary thyroid carcinoma. In the case of calcified papillary thyroid carcinoma, the occurrence of central lymph node metastasis may be associated with tumor classification 27,28.

In ultrasonic elastography, the hardness of tissues is assessed by measuring Young's modulus value, with clear two-dimensional longitudinal sections and the elasticity imaging mode used, and tumor lesions selected as the region of interest by sampling frames. The larger the value is, the smaller the tissue deformation and the greater the hardness will be. Ultrasonic elastography is extensively applied in diagnosing diseases of soft tissues such as breast, thyroid and liver 29,30. The results of the present study uncovered that the Eavg in the metastasis group was higher than that in the non-metastasis group, and high-level Eavg acted as a risk factor for central lymph node metastasis of papillary thyroid carcinoma. Pathological studies have denoted that the differentiation and proliferation of malignant tumor cells are often accompanied by fibrosis, which will enhance the hardness and invasiveness of tumor lesions, thus resulting in lymph node metastasis. In other words, papillary thyroid carcinoma lesions with a high Eavg value are harder and more likely to develop central lymph node metastasis 31,32.

In CEUS, a clear section is selected and fixed, and the perfusion of contrast agents in lesions is observed to assess the pathological condition 33. RT refers to the time required for the signal to rise to the final value, and PI is an important indicator for evaluating tumor angiogenesis, which can accurately reflect tumor angiogenesis 34. It was discovered in the present study that RT was shorter in the metastasis group than that in the non-metastasis group, while PI was higher in the metastasis group than that in the non-metastasis group, and low-level RT and high-level PI were risk factors for central lymph node metastasis of papillary thyroid carcinoma. This is attributed to the fact that central lymph node metastasis has a close relation to the blood supply to papillary thyroid carcinoma, and richer blood supply indicates a higher risk of lymph node metastasis 35. The tumors without lymph node metastasis have relatively less new blood vessels formed therein, and the vessels therein are smaller with lower density, so the effective vessel area is smaller. Accordingly, the contrast agent enters slowly, manifested as longer RT and lower PI 36,37. In contrast, many new vascular networks are formed in tumors with lymph node metastasis, so that the contrast agent can reach the final value quickly during CEUS, manifested as shorter RT and higher PI value.

In this study, an ultrasound-based radiomics model for prediction was constructed, and the equation was Logit(P)=2.320+1.323× maximum lesion diameter +3.558× echo +3.131× margin +2.470× capsule invasion +2.459× calcification +1.072 × Eavg +1.074 × RT +1.224× PI. According to the ROC curve plotted, the AUC of this prediction model for predicting central lymph node metastasis of papillary thyroid carcinoma was 0.889, demonstrating that this prediction model has high stability and predictive value.

Nevertheless, this study is limited. The sample size is small, which may easily lead to overfitting. Further studies with larger sample sizes are ongoing in our group to confirm our findings.

## Conclusion

In conclusion, the central lymph node metastasis of papillary thyroid carcinoma may be related to ultrasound imaging features/parameters such as the maximum lesion diameter, hypoechoic/extremely hypoechoic, lobulated or irregular margin, capsule invasion, microcalcification/macrocalcification or peripheral calcification, high-level Eavg, low-level RT, and high-level PI. The established ultrasound-based radiomics model can be employed for early identifying the central lymph node metastasis of papillary thyroid carcinoma.

## Figures and Tables

**Table 1:** General data.

Indicator		Metastasis group (n=59)	Non-metastasis group (n=67)	*t*	P
Gender	Male	17 (28.81)	24 (35.82)	*χ*^2^=0.702	0.402
	Female	42 (71.19)	43 (64.18)		
Age [(‾*x* ± s), year]		47.34±9.16	46.83±8.54	*t*=0.323	0.747
Body mass index [(‾*x* ± s), kg/m^2^]		25.36±1.97	25.51±2.03	*t*=0.420	0.676
Hemoglobin [(‾*x* ± s), ×g/L]		119.83±15.49	121.38±16.34	*t*=0.544	0.587
Platelet count [(‾*x* ± s), ×10^9^/L]		204.36±34.82	198.73±36.57	*t*=0.882	0.380
White blood cell count [(‾*x* ± s), ×10^9^/L]		9.53±3.04	9.61±3.12	*t*=0.145	0.885

**Table 2:** Routine ultrasonography results of metastasis and non-metastasis groups.

Indicator		Metastasis group (n=59)	Non-metastasis group (n=67)	Statistical value	P
Maximum lesion diameter [(‾*x* ± s), mm)		13.08±3.64	9.54±3.15	*t*=5.852	0.000
Number of nodules [n (%)]	Single	19 (32.20)	21 (31.34)	*χ*^2^=0.011	0.918
	Multiple	40 (67.80)	46 (68.66)		
Aspect ratio of lesions [n (%)]	≥1	41 (69.49)	39 (58.21)	*χ*^2^=1.723	0.189
	<1	18 (30.51)	28 (41.79)		
Location [n (%)]	Right lobe	30 (50.85)	32 (47.76)	*χ*^2^=2.862	0.239
	Left lobe	25 (42.37)	34 (50.75)		
	Isthmus	4 (6.78)	1 (1.49)		
Echo [n (%)]	Hyperechoic or isoechoic	8 (13.56)	24 (35.82)	*χ*^2^=8.206	0.004
	Hypoechoic/extremely hypoechoic	51 (86.44)	43 (64.18)		
Margin [n (%)]	Smooth	31 (52.54)	52 (77.61)	*χ*^2^=8.771	0.003
	Lobulated or irregular	28 (47.46)	15 (22.39)		
Capsule invasion [n (%)]	Yes	22 (37.29)	13 (19.40)	*χ*^2^=5.002	0.025
	No	37 (62.71)	54 (80.60)		
Calcification [n (%)]	No	31 (52.54)	49 (73.13)	*χ*^2^=9.982	0.007
	Microcalcification	24 (40.68)	18 (26.87)		
	Macrocalcification or peripheral calcification	4 (6.78)	0		
Blood flow distribution [n (%)]	Grade 0	6 (10.17)	12 (17.91)	*χ*^2^=2.960	0.398
	Grade I	17 (28.81)	16 (23.88)		
	Grade Ⅱ	33 (55.93)	38 (56.72)		
	Grade III	3 (5.08)	1 (1.49)		

**Table 3:** Real-time SWE results of metastasis and non-metastasis groups (‾*x* ± s).

Indicator	Metastasis group (n=59)	Non-metastasis group (n=67)	*t*	P
Eavg (kPa)	48.52±9.64	36.21±11.37	6.507	0.000
Eratio	1.62±0.39	1.73±0.41	1.537	0.127

**Table 4:** CEUS results of metastasis and non-metastasis groups (‾*x* ± s).

Indicator	Metastasis group (n=59)	Non-metastasis group (n=67)	*t*	P
RT (s)	6.19±2.14	7.78±2.06	4.512	0.000
TTP (s)	20.41±9.43	20.50±10.57	1.537	0.127
PI (dB)	15.03±4.52	10.29±4.73	5.731	0.000
AUCt (dBs)	396.82±118.39	421.53±126.71	1.126	0.262

**Table 5:** Diagnostic values of ultrasound imaging features/parameters for central lymph node metastasis.

Test variable	AUC	Standard error	P	95% confidence interval		Cut-off value	Sensitivity	Specificity	Youden index
				Lower limit	Upper limit				
Maximum lesion diameter	0.773	0.042	0.000	0.690	0.856	10.960 mm	0.763	0.642	0.405
Echo	0.579	0.051	0.128	0.479	0.679	-	0.695	0.537	0.232
Margin	0.616	0.051	0.025	0.517	0.715	-	0.559	0.791	0.350
Capsule invasion	0.589	0.051	0.084	0.489	0.690	-	0.627	0.806	0.433
Calcification	0.603	0.051	0.047	0.503	0.703	-	0.525	0.731	0.256
Eavg	0.687	0.047	0.000	0.595	0.778	40.805 kPa	0.610	0.627	0.237
RT	0.551	0.052	0.320	0.451	0.652	7.350 s	0.576	0.710	0.286
PI	0.740	0.044	0.000	0.654	0.826	11.920 dB	0.712	0.642	0.354
Combination	0.817	0.037	0.000	0.745	0.890	-	0.847	0.687	0.534

**Table 6:** Results of multivariate logistic regression analysis on central lymph node metastasis.

Independent variable	*β*	Standard error	*Wald χ^2^*	P	*Odds ratio*	95% CI
Maximum lesion diameter (original value)	0.303	0.064	22.171	0.000	1.323	1.193-1.535
Echo (with hyper-echo or iso-echo as reference)	1.269	0.458	7.688	0.006	3.558	1.451-8.727
Margin (with smooth margin as reference)	1.141	0.392	8.467	0.004	3.131	1.451-6.755
Capsule invasion (with no invasion as reference)	0.904	0.410	4.868	0.027	2.470	1.106-5.514
Calcification (with no calcification as reference)	0.900	0.379	5.623	0.018	2.459	1.169-5.172
Eavg (original value)	0.069	0.019	13.266	0.000	1.072	1.033-1.112
RT (original value)	0.071	0.063	1.295	0.255	1.074	0.950-1.214
PI (original value)	0.202	0.047	18.569	0.000	1.224	1.116-1.341
Constant	2.320	0.594	15.257	0.000	10.171	-

**Table 7:** Validation of model prediction effect.

AUC	Standard error	P	95% CI		Sensitivity	Specificity	Youden index
			Lower limit	Upper limit			
0.889	0.029	0.000	0.832	0.946	0.898	0.731	0.629

## Abbreviations

ATA: American Thyroid Association
AUC: area under the curve
AUCt: area under TIC
CEUS: contrast-enhanced ultrasonography
CI: confidence interval
PI: peak intensity
ROC: receiver operating characteristic
RT: rising time
SWE: shear wave elastography
TIC: The time-intensity curve
TTP: time to peak
